# Study on the Effect of Electrolytes on Processing Efficiency and Accuracy of Titanium Alloy Utilizing Laser and Shaped Tube Electrochemical Machining

**DOI:** 10.3390/ma17030689

**Published:** 2024-01-31

**Authors:** Chenyu Sun, Yufeng Wang, Yong Yang, Zhehe Yao, Yunfeng Liu, Qiang Wu, Jie Yan, Jianhua Yao, Wenwu Zhang

**Affiliations:** 1 College of Mechanical Engineering, Zhejiang University of Technology, Hangzhou 310023, China; sunchenyu@nimte.ac.cn (C.S.); zhyao@zjut.edu.cn (Z.Y.); liuyf76@126.com (Y.L.); laser@zjut.edu.cn (J.Y.); 2 Ningbo Institute of Materials Technology and Engineering, Chinese Academy of Sciences, Ningbo 315201, China; yangyong1994@nimte.ac.cn (Y.Y.); zhangwenwu@nimte.ac.cn (W.Z.); 3 Suzhou Electric Machining Machine Tool Research Institute Co., Ltd., Suzhou 215011, China; 18962175591@163.com (Q.W.); jeoyan5100@163.com (J.Y.)

**Keywords:** laser and electrochemical machining, passivating electrolyte, aggressive electrolyte, material removal mechanisms, side gap

## Abstract

Electrochemical machining (ECM) has become more prevalent in titanium alloy processing. However, the presence of the passivation layer on the titanium alloys significantly impacts the performance of ECM. In an attempt to overcome the passivation effects, a high-temperature electrolyte or the addition of halogen ions to the electrolyte has been used. Still, it often results in compromised machining accuracy and surface roughness. This study applied laser and shaped tube electrolytic machining (Laser-STEM) for titanium alloy drilling, where the laser was guided to the machining zone via total internal reflection. The performance of Laser-STEM using different types of electrolytes was compared. Further, the effects of laser power and pulse voltage on the machining side gap, material removal rate (MRR), and surface roughness were experimentally studied while drilling small holes in titanium alloy. The results indicated that the use of passivating electrolytes improved the machining precision, while the MRR decreased with an increase in laser power during Laser-STEM. The MRR showed an increase while using aggressive electrolytes; however, at the same time, the machining precision deteriorated with the increase in laser power. Particularly, the maximum feeding rate of 6.0 mm/min for the tool electrode was achieved using NaCl solution as the electrolyte during Laser-STEM, marking a 100% increase compared to the rate without the use of a laser. Moreover, the model and equivalent circuits were also established to illustrate the material removal mechanisms of Laser-STEM in different electrolytes. Lastly, the processing of deep small holes with a diameter of 1.5 mm, a depth of 38 mm, and a surface roughness of Ra 2 µm was achieved via Laser-STEM without the presence of a recast layer and heat-affected zones. In addition, the cross-inner flow channels in the titanium alloys were effectively processed.

## 1. Introduction

With the continuous improvement in the performance of advanced aerospace engines, there is a growing utilization of lightweight materials with high-temperature resistance and excellent mechanical properties. In this regard, titanium alloys have gained significant popularity in aero-engineering, contributed by their superior properties, such as low density, high specific strength, and high temperature and corrosion resistance [1]. The incorporation of titanium alloys allows for significant weight reduction in components while simultaneously improving their strength. The amount of titanium alloys utilized has emerged as an important indicator for assessing the advancement of next-generation aero engines [2]. As a result, there is a pressing demand for the machining of key components and structures made of titanium alloys. However, titanium alloys suffer from certain drawbacks, such as poor room-temperature plasticity, toughness, and thermal conductivity, leading to their subpar mechanical machining performance. This, in turn, leads to issues of shortened tool lifespan and high costs. These processing difficulties have limited the broader applications of titanium alloy in aero engines [2]. Therefore, addressing the issue of processing titanium alloy efficiently, cost-effectively, and with high surface integrity remains of paramount importance.

Small hole drilling is crucial for the functionalization of titanium alloys, presenting a challenge to the existing processes [3,4]. In mechanical-based methods, the occurrence of burrs in the orifice is a common issue [5,6,7]. Moreover, when dealing with small holes with a diameter smaller than 1 mm, tool vibration can lead to poor processing accuracy and tool breakage [8,9]. Thermal-based processes, such as electrical discharge machining and laser beam processing, are capable of efficiently removing material using instantaneous high temperatures. However, these processes suffer from issues such as the formation of recast layers, micro-cracks, and heat-affected zones [2]. Additionally, femtosecond pulsed laser processing also encounters difficulties in altogether avoiding surface thermal damage while processing small holes with a large depth-to-diameter ratio [10].

Meanwhile, electrochemical machining (ECM) is not limited by the mechanical properties of the conductive material and can achieve high surface integrity without experiencing tool wear [11,12]. Unlike other methods, ECM also has the ability to avoid the formation of both the recast layers and heat-affected zones [13]. Consequently, ECM has been increasingly adopted to process titanium alloys. Along these lines, Chen et al. investigated the electrochemical dissolution characteristics of titanium alloys with different phases, achieving a surface roughness of Ra 0.28 µm through ECM [11]. Similarly, Xu et al. verified the feasibility of the electrochemical dissolution of titanium alloy, revealing a surface roughness of Ra 0.37 µm, along with a removal rate of 3.1 mm^3^/A min [13]. Additionally, Xu et al. optimized the ECM parameters and successfully processed titanium alloy fan blades at a tool electrode feeding rate of 1.2 mm/min, resulting in a profile accuracy of 0.07 mm [14]. In a study by Klock et al., it was demonstrated that the machined edges of titanium alloys exhibited no heat-affected zone and no diffusion of foreign atoms [15]. These findings conclude that titanium alloys can be processed using ECM, resulting in high surface quality.

Shaped tube electrolytic machining (STEM), a variation of ECM, uses a metal tube as the tool electrode, allowing for deep-hole processing. Hung et al. relieved the effects of stray currents during STEM by applying an insulating layer on the sidewall of the tube electrode, which reduced the difference between the entrance and exit of the hole, leading to improved drilling accuracy [16]. Sakamotoa et al. found that the hole shape accuracy deteriorated when the electrolyte flow rate was decreased during ECM, which was attributed to the difficulties in flowing the electrolytic products away from the machining gap [17]. In a separate study, Jaina et al. employed a tubular tool electrode to machine a tapered hole with a diameter of 100 µm on the surface of a titanium alloy [18]. Furthermore, Liu et al. proposed a novel tube electrode structure consisting of an insulating substrate and a conductive film, which was used to process microvias with a diameter of 178 µm and a depth-to-diameter ratio of 3 [19]. In another attempt, Perla et al. applied Taguchi’s experimental design method to analyze the impact of voltage and the duty cycle on tool tube electrode processing and revealed that the duty cycle had a more significant effect on surface roughness, while the applied voltage greatly affected dimensional deviation [20]. Meanwhile, Geethapiriyan et al. performed experiments on titanium alloys using STEM and showed a 17% reduction in machining roughness and a 5.3% increase in material removal rate for nickel-plated tube electrodes compared to unplated tube electrodes [21]. In another interesting study, Anuj et al. indicated that adding sodium hydroxide to sodium chloride and sodium nitrate solutions resulted in a reduction in the hole roundness error [22]. Zhang et al. confirmed that the utilization of an external rinse solution around the machining area effectively decreased the radius of the stray corrosion-affected area [23]. Bian et al. investigated the effect of cathode materials on the electrolytic machining of stainless steel with tool tube electrodes and experimentally concluded that the use of 6061 aluminum alloy effectively mitigated the stray current corrosion during the machining process and reduced the hole taper [24]. Özerkan designed a device for processing small diameter holes in powder metal steel, which achieved a 65% reduction in electrolyte consumption by rotating the tool tube electrode while simultaneously enhancing the efficiency of debris discharge from the side machining gap [25]. In order to improve the efficiency of titanium alloy drilling via STEM, it is necessary to address passivation by using methods that utilize high-temperature electrolytes (>30 °C) or introduce halogen ions like Cl^–1^ and Br^–1^ into the electrolyte. However, utilizing these methods may result in compromised machining accuracy and surface roughness. Additionally, the distribution of electric current density may lead to the formation of a protrusion in the central machining zone, hindering the diffusion rate of electrolytic products and deteriorating the machining stability. Consequently, when drilling titanium alloys using STEM, the feeding rate of a tubular electrode is typically kept below 2.0 mm/min to prevent electric short circuits between the tubular electrode and workpiece [26,27]. Thus, there is a need to further enhance the electrochemical machining efficiency of titanium alloy drilling while simultaneously preserving machining precision.

In the present study, a combination of laser and shaped tube electrolytic machining (Laser-STEM) was adopted for drilling titanium alloys, where the laser was guided to the machining zone via total internal reflection. Two types of electrolytes were utilized in this study, including aggressive and passivating electrolytes. The performance of Laser-STEM was assessed using different kinds of electrolytes. In addition, the experimental investigation focused on examining the effects of laser power, voltage, and the tool electrode feeding rate on machining efficiency, accuracy, and surface roughness. Furthermore, the study explored the different material removal mechanisms that occur when processing titanium alloys with Laser-STEM using different types of electrolytes.

## 2. Materials and Methods

### 2.1. Principle of Laser-STEM

**Figure 1:**
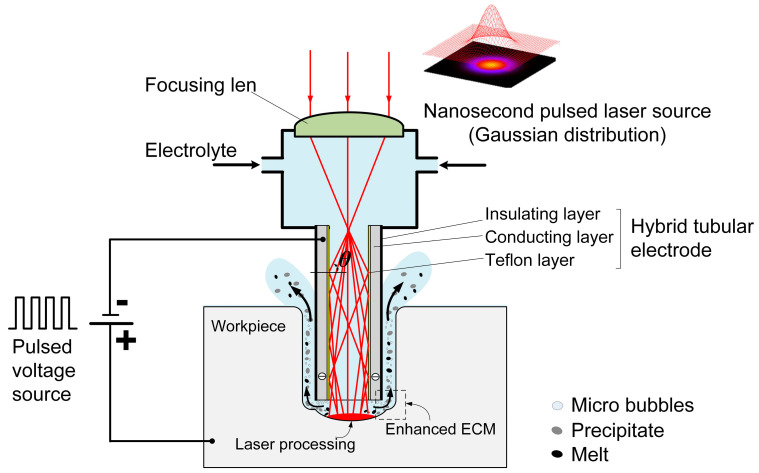
Schematic illustration of the hybrid Laser-STEM process.

Laser-STEM utilizes total internal reflection to guide the laser beam toward the machining zone. As shown in Figure 1, the laser is focused at the entrance of the tubular electrode using a focusing lens. The tubular electrode comprises a conducting layer and a Teflon layer with a low optical refractive index (*n*_2_ = 1.24), which is smaller than the refractive index of the electrolyte (*n*_1_ = 1.35). When the incident angle *θ* at the interface of the electrolyte/Teflon layer exceeds a critical value of arcsin (*n*_1_/*n*_2_), total internal reflection occurs at the interface. As a result, the laser can effectively transmit to the machining zone with an efficiency greater than 70% [28].

In Laser-STEM, the laser is directed to the central machining area through the inner hole of the tubular electrode. By feeding the tubular electrode, the laser can be transmitted to the large-depth machining zone, making it suitable for deep-hole drilling applications. When the laser intensity surpasses a threshold value, the workpiece materials can be effectively removed through laser processing. The materials in both the central machining zone and the side machining gap can be synchronously removed via ECM. Employing ECM at the side machining gap guarantees the attainment of the high surface quality of the processed microholes. Meantime, the laser-induced temperature rise leads to an increase in the electrolyte temperature within the processing area, thereby improving the ECM rate. During Laser-STEM, an electrolyte is flushed to the machining area from the inner hole of the tubular electrode and subsequently flushed out from the side gap between the tubular electrode and the workpiece, enabling the effective removal of microbubbles, electrolytic products, plasma, melt, and heat from the machining gap. Therefore, Laser-STEM allows the processing of holes with greater depth while maintaining high surface quality.

### 2.2. Experimental Setup

**Figure 2:**
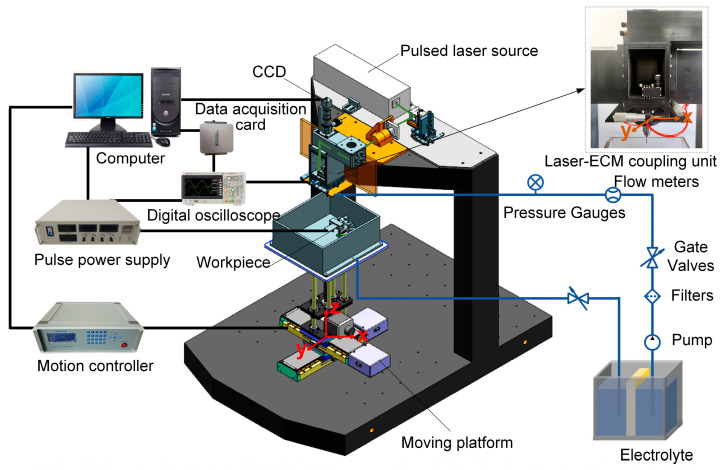
The experimental setup developed for Laser-STEM.

Figure 2 illustrates the schematic diagram of the experimental setup developed for Laser-STEM, consisting of a laser power source, an optical guide system, an ECM system, a liquid transportation system, a laser-ECM coupling unit, a motion control system, and a data acquisition system.

The pulsed laser emitted from the laser generator is directed toward the entrance of the tubular electrode using reflective mirrors and a focusing lens. In this setup, a pulsed laser source with a wavelength of 532 nm, a pulse duration of 16.3 ns, and a pulse repletion frequency of 8 kHz is used. The laser beam is focused at the entrance of the tube electrode through the use of a focusing lens with a focal length of 100 mm. The position of the laser focal point and the entrance of the tubular electrode are continuously monitored using a CCD monitoring system. The electrolyte supply system uses a diaphragm pump to deliver electrolytes to the laser-ECM coupling unit. After the machining process, the electrolyte flows into a waste tank, where it can undergo settling and filtering before being reused for further operations. The metal tube in the tubular electrode and the workpiece are connected to the positive and negative polarities of a pulse power supply, respectively, operating at a pulse frequency of 20 kHz and a duty ratio of 50%. The machining current is acquired and analyzed in real-time, using a data acquisition card (PCI-6221, NI) to monitor the status of the Laser-STEM. The electrolytic cell used for securing the workpiece is mounted on an X–Y–Z motion platform, which offers a travel range of 250 mm × 250 mm × 250 mm and a motion accuracy of 0.001 mm.

### 2.3. Materials and Measurement

A polished specimen made of TC4 titanium alloy with dimensions of 50 mm × 50 mm × 2 mm was utilized in the experiments. Before conducting experiments, the material underwent a cleaning process in an ultrasonic cleaning machine for 10 min using anhydrous ethanol. The electrolytes employed in this study included sodium nitrate (NaNO_3_) and sodium chloride (NaCl), with NaNO_3_ serving as the aggressive electrolyte and NaCl as the passivating electrolyte. A brass tube with an outer diameter of 1.25 mm and an inner diameter of 0.8 mm was used to prepare the tubular electrode. A Teflon layer was installed inside the tube, while an insulating layer was applied to the outside wall of the tube. Following the completion of the experiments, the specimen was immersed in anhydrous ethanol and ultrasonically cleaned for 30 min.

Before the processing of the specimen, its weight was measured using an electric balance (EX125ZH, Ohaus, Parsippany, NJ, USA) with a resolution of 0.001 mg. Once the experiments were completed, the workpiece was weighed again to determine the quality of the material after processing. To ensure accuracy, three holes were machined using each set of machining parameters, and the average value of the three holes was taken into consideration. The material removal rate (MRR) was determined by calculating the ratio of the mass of material removed to the processing time, which can be expressed as:

(1) $$\mathrm{MRR}=\frac{m_{0}-m_{a}}{t}$$

where *m*_0_ represents the mass of the material before processing, *m_a_* represents the mass of the material after processing, and *t* denotes the processing time. The machined hole was measured using a laser confocal microscope (VK-X200K, Keyence, Tokyo, Japan). Furthermore, a scanning electron microscope (SEM, SU5000, Hitachi, Tokyo, Japan) along with an energy dispersive spectrometer (EDS) was used to examine the microscale morphology and element distribution of the machined surface. The machining accuracy was quantified using the lateral/side machining gap (Δ*_s_*), which can be expressed as:

(2) $$\Delta_{s}=\frac{D_{a}-D}{2}$$

where *D*_a_ denotes the average hole diameter, and *D* signifies the outer diameter of the tubular electrode. The front machining gap (Δ*_b_*) is defined as the difference between the hole depth and the feed depth and can be calculated using the following expression:

(3) $$\Delta_{b}=h_{a}-(h-\Delta_{0})$$

where *h*_a_ represents the distance from the workpiece surface to the bottom of the hole, *h* denotes the tube electrode feeding depth, and Δ_0_ is the initial front machining gap.

## 3. Results

### 3.1. Observing Phenomena in the Machining Gap during Laser-STEM

**Figure 3:**
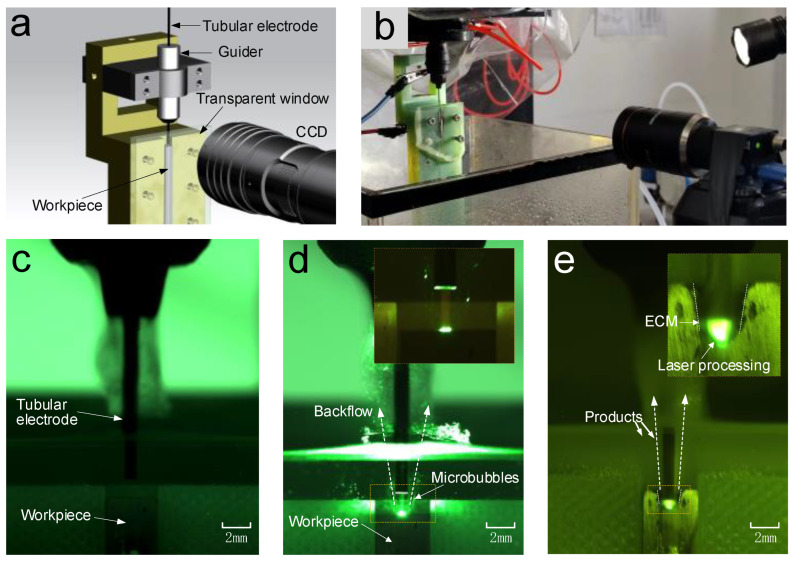
Observing phenomena in the gap during Laser-STEM: (**a**) the 3D model of the experimental apparatus, (**b**) a photograph of the experimental apparatus, (**c**) the initial stage without laser, (**d**) laser acting on the workpiece through the inner hole of the tubular electrode, and (**e**) the phenomena occurring in the machining gap during the hole drilling process.

An experimental apparatus was designed to observe the phenomena occurring in the machining gap through a transparent glass window, as shown in Figure 3a,b. A semi-circular flow channel was specifically machined on the glass. Simultaneously, a semi-circular hole was processed on the titanium alloy workpiece, which was then affixed to the glass window. The CCD camera was employed to observe and capture the phenomena taking place within the machining gap.

Figure 3c shows the initial stage of Laser-STEM, where the hybrid tubular electrode was positioned above the workpiece, maintaining an initial interelectrode gap. As the laser was transmitted through the tubular electrode, it interacted with the workpiece through the electrolyte within the front machining gap, as shown in Figure 3d. During the laser processing, microbubbles were generated at the laser-processed area and were then expelled from the machining zone by the inner electrolyte jet originating from the inner hole of the tubular electrode. Meanwhile, this process effectively removed electrolytic products, laser-induced plasma, and heat from the machining zone. As the tubular electrode was fed toward the workpiece, the laser could penetrate into the large-depth machining area, as depicted in Figure 3e. The workpiece materials located at the front machining gap were removed through the combined effects of laser processing and ECM. On the other hand, within the side machining gap, the materials were primarily removed using ECM.

### 3.2. Comparison of Laser-STEM Performance with Different Types of Electrolytes: Effect of Laser Power

To study the effects of laser power and electrolyte, a series of experiments were conducted with varying laser powers. The experimental setup included a feeding distance of 0.9 mm, an initial front machining gap of 0.4 mm, a voltage of 20 V, and laser powers ranging from 0 to 5 W.

**Figure 4:**
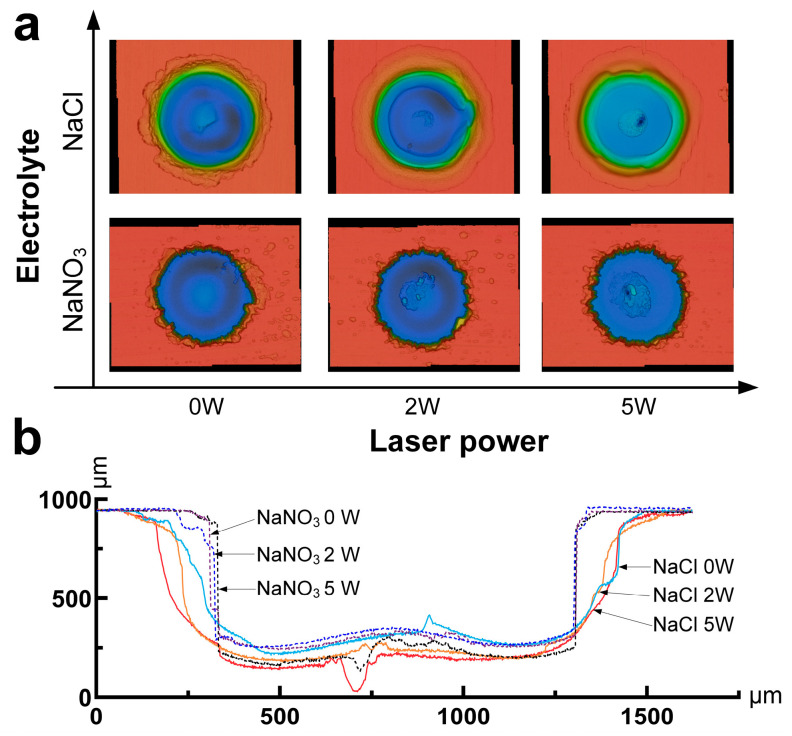
Comparison of the processed microstructures with varying laser power and different types of electrolytes: (**a**) three-dimensional morphology of the microstructures; (**b**) the cross-sectional profiles processed in different conditions.

Figure 4 presents a comparison of the processed microstructures with different laser power settings and various electrolytes. The microcavities processed using NaNO_3_ electrolyte exhibited a rougher entrance, likely attributed to the formation of a passivation layer on the surface. Conversely, the microcavities processed with NaCl electrolyte displayed a more rounded entrance, characterized by evident entrance rounding. With the increase in laser power, the occurrence of stray corrosion on the surface of the workpiece processed with NaNO_3_ gradually decreased, ascribing to the increasing effects of the passivation layer. By comparing the morphology of the hole bottom, it was revealed that, at a laser power of 2 W, no significant laser crater was observed, indicating weak laser removal ability. However, at a power of 5 W, a laser crater was generated due to the higher laser intensity exceeding the removal threshold of titanium alloys.

**Figure 5:**
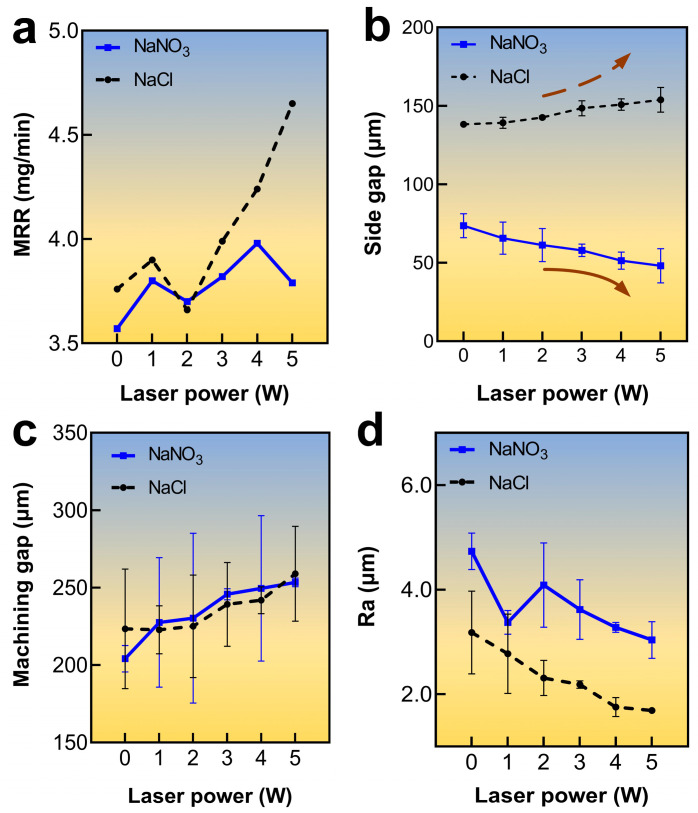
Variation of (**a**) MRR, (**b**) side gap, (**c**) machining gap, and (**d**) surface roughness with laser power.

**Figure 6:**
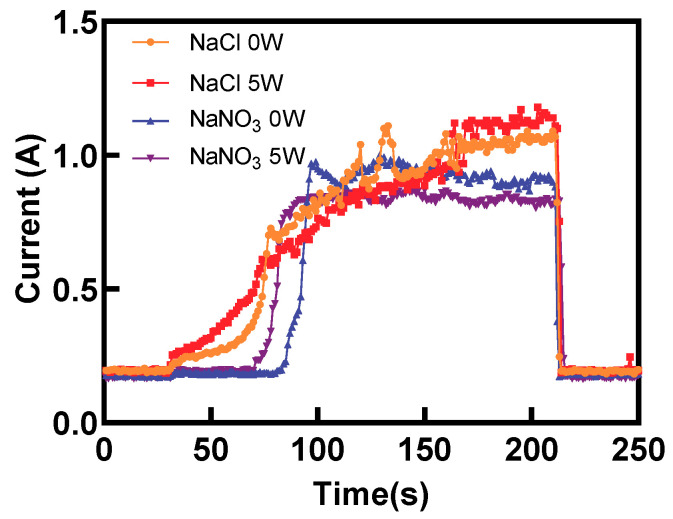
Variation of electric current with time using NaNO_3_ and NaCl electrolytes at laser powers of 0 W and 5 W.

Figure 5a shows the impacts of laser power on the MRR. Notably, when the NaCl electrolyte was used, the MRR fluctuated around 3.8 mg/min within the 0–2 W laser power range. Meanwhile, as the laser power increased, the MRR exhibited an upward trend, rising from 3.99 mg/min to 4.65 mg/min. This could be attributed to the laser-induced temperature increase in the electrolyte, which increased the electric current efficiency. Next, when the laser power was increased from 0 W to 5 W, a noticeable rise in the electric current was observed, subsequently enhancing the rate of ECM, as shown in Figure 6. At lower laser power levels, the laser-induced removal of titanium alloy was minimal. Surprisingly, the temperature-rise effect of the laser played a dominant role in enhancing the ECM rate. The localized laser-induced temperature of the electrolyte increased the electric current density for ECM and the rate of passivation layer generation, resulting in slight fluctuations in MRR. When the laser power was increased to 3–5 W, the laser power density exceeded the material removal threshold of titanium alloy. At this range, the laser not only provided a temperature rise effect but also directly removed the material, leading to a substantial increase in MRR. Contrarily, the MRR demonstrated an increasing trend when a NaNO_3_ electrolyte was used. However, when the laser power was raised to 5 W, the MRR showed a slight decrease, which was due to the laser-induced thermal effect on the electrolyte when using the passivating electrolyte, which promoted the formation rate of the passivation layer. Consequently, the electric current density during Laser-STEM was reduced, leading to a slight decline in the MRR. As shown in Figure 6, the electric current exhibited a decrease when the laser power was set at 5 W compared to the case without the laser. This reduction in electric current resulted in a lower ECM rate. Furthermore, increasing the laser power to 5 W led to an increase in the thickness of the passivation layer, causing a decrease in MRR.

Figure 5b,c displays the effects of laser power on machining accuracy and the front machining gap for both types of electrolytes. The results indicated that the side gap and front machining gap in the NaCl solution exhibited a gradual increase as the laser power was increased. Notably, the side gap after NaCl processing increased from 164.15 µm to 178.82 µm, while the front machining gap increased from 222.78 µm to 258.93 µm as the laser power increased from 0 W to 5 W. Conversely, when using a NaNO_3_ electrolyte, the side gap showed a decreasing trend, whereas the front machining gap demonstrated an increasing trend, with an increase in laser power. The side gap of the sodium nitrate solution decreased from 90.66 μm to 73.08 μm when the laser power was increased from 0 W to 5 W. The removal effect of the laser increases the speed of end material removal. Moreover, the laser-induced thermal effects promoted the formation of the passivation layer on the surface of the titanium alloys when using NaNO_3_ electrolytes in Laser-STEM, which, in turn, reduced stray current corrosion and enhanced the machining precision of ECM.

**Figure 7:**
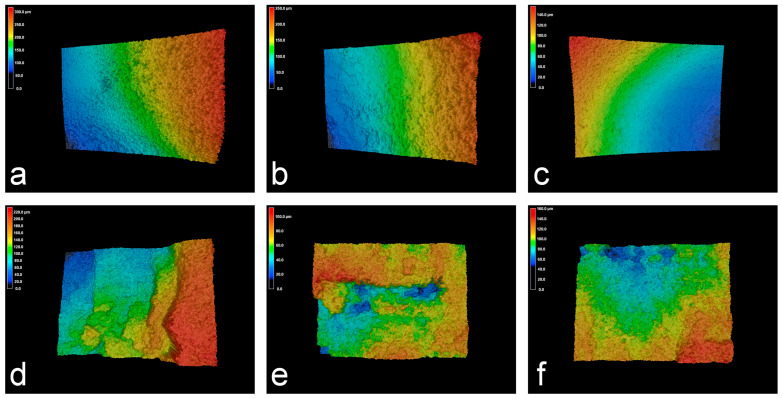
The 3D morphologies of the sidewall of the microstructures processed using Laser-STEM at different conditions: (**a**) NaCl, voltage 20 V, laser power 0 W; (**b**) NaCl, voltage 20 V, laser power 2 W; (**c**) NaCl, voltage 20 V, laser power 5 W; (**d**) NaNO_3_, voltage 20 V, laser power 0 W; (**e**) NaNO_3_, voltage 20 V, laser power 2 W; (**f**) NaNO_3_, voltage 20 V, laser power 5 W.

Figure 5d shows the influence of laser power on sidewall surface roughness for both types of electrolytes. With the increase in laser power, the surface roughness of the specimens processed with NaNO_3_ electrolyte decreased from Ra 4.37 µm to Ra 3.03 µm, while it decreased from Ra 2.77 µm to Ra 1.69 µm in the case of the NaCl electrolyte, suggesting a decrease in surface roughness value in both the electrolytes. However, the surface appeared smoother when the NaCl electrolyte was used, as shown in Figure 7. In contrast, the use of the NaNO_3_ electrolyte resulted in a larger surface roughness value, primarily due to the formation of the passivation layer on the surface. Therefore, increasing laser power can improve the processing efficiency and surface quality in Laser-STEM. Specifically, when using an oxidizing electrolyte like NaNO_3_, the higher laser power was beneficial for the accuracy of ECM. On the other hand, when employing an aggressive electrolyte such as NaCl, increasing the laser power can improve the efficiency of ECM.

### 3.3. Influence of Voltage on the Performance of Laser-STEM

To study the influence of voltage on the performance of Laser-STEM for titanium alloys, a series of experiments were carried out with varying voltages. The experimental conditions included a feeding distance of 0.9 mm, an initial machining gap of 0.4 mm, and a constant laser power of 5 W.

**Figure 8:**
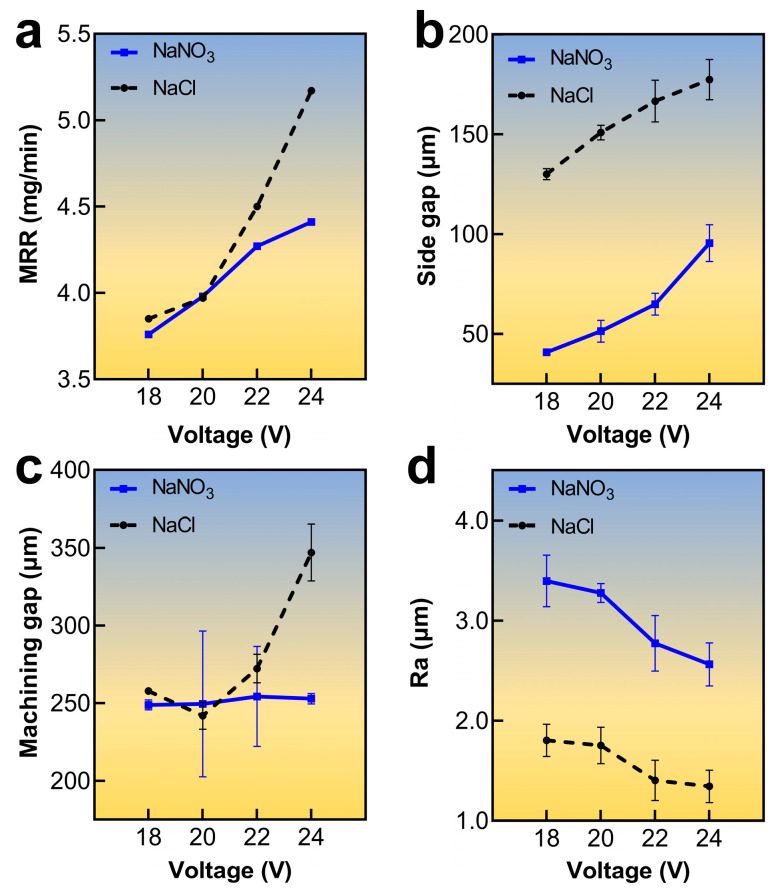
Variation of (**a**) MRR, (**b**) side gap, (**c**) machining gap, and (**d**) surface roughness with voltage.

As depicted in Figure 8a, with an increase in voltage from 18 V to 24 V, the MRR was increased. For the NaNO_3_ solution, the MRR increased from 3.76 mg/min to 4.41 mg/min, whereas, for the NaCl solution, the MRR increased from 3.85 mg/min to 5.17 mg/min. Notably, the MRR obtained with the NaNO_3_ solution remained lower compared to that with the NaCl electrolyte. While the laser was capable of removing the passivation layer at the center area of the hole bottom and improving the electrolytic capability of the NaNO_3_ solution to some extent, it was unable to eliminate the passivation layer at the periphery of the hole bottom and the hole wall. Contrarily, the chloride ions in the NaCl solution effectively destroyed the passivation layer, leading to a higher current density and MRR. As a result, a higher MRR could be obtained using NaCl electrolyte in Laser-STEM.

Next, Figure 8b,c illustrates the effect of voltage on machining accuracy and the machining gap for both types of electrolytes during Laser-STEM. The results showed that the side machining gap increased the voltage for both electrolytes, implying a deterioration in the machining precision of Laser-STEM with the increase in voltage. As the materials were mainly removed using the ECM process, the side gap became more prominent due to the enhanced MRR of ECM. Moreover, it was observed that the side gap of the Laser-STEM using the NaNO_3_ electrolyte was smaller compared to that when using the NaCl electrolyte. The formation of the passivation layer on the surface of titanium alloys while using the NaNO_3_ electrolyte could relieve the effects of stray current corrosion, thereby improving the machining efficiency of Laser-STEM. The machining gap of the NaCl solution exhibited an upward trend with higher processing voltage; moreover, it remained stable at approximately 250 µm in the case of the NaNO_3_ solution. The presence of the passivation layer caused by the NaNO_3_ electrolyte hindered the MRR in the axial direction within the voltage range. Thus, the utilization of the NaNO_3_ electrolyte in Laser-STEM was not conducive to increasing the feeding rate of the tubular electrode.

Furthermore, Figure 8d shows the influence of voltage on side-wall surface roughness. As the voltage increased, the surface became coarser for both electrolytes. Specifically, the surface roughness decreased from Ra 3.39 µm to Ra 2.56 µm with increasing voltage in the case of the NaNO_3_ solution, whereas it decreased from Ra 1.8 µm to Ra 1.34 µm when the voltage was increased in the NaCl electrolyte. The larger reduction in the machined surface roughness when using the NaNO_3_ electrolyte could be ascribed to the surface pitting and flaking on the passivation layer formed during the machining process.

### 3.4. Determination of Maximum Feeding Rate Using Different Types of Electrolytes

**Figure 9:**
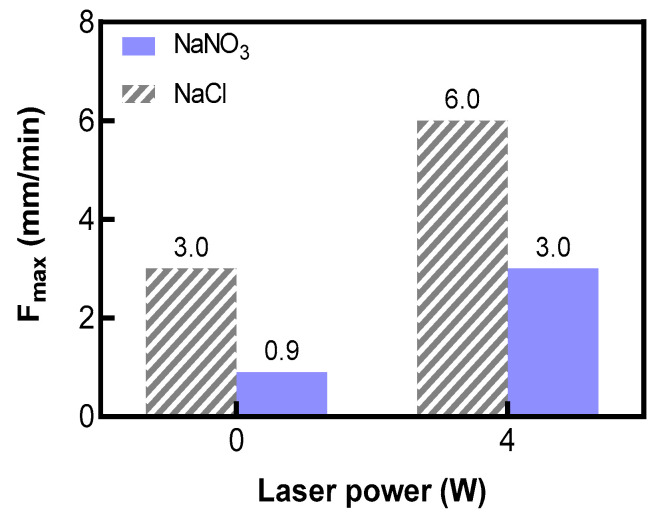
Comparison of the maximum feeding rate of the tubular electrode for NaCl and NaNO_3_ electrolytes and laser power.

To determine the maximum stable machining speed of Laser-STEM, experiments were conducted using a voltage of 20 V, a laser power of 4 W, and an initial machining gap of 0.4 mm. The maximum stable machining speed was examined using the obtained maximum feeding rate of the tubular electrode without the occurrence of electric short circuits. Figure 9 presents the comparison of the maximum feeding rate for the two types of electrolytes. It was observed that the maximum stable feed rate for the NaCl solution increased from 3 mm/min to 6 mm/min; on the other hand, it increased from 0.9 mm/min to 3 mm/min for the NaNO_3_ solution. The thermal effects of the laser led to an increase in the electric current density and ECM rate, which, in turn, raised the machining rate in the front and side gaps. Additionally, the direct removal of material by the laser speeds up the MRR, consequently increasing the upper limit of the tube electrode feed rate. However, as the feeding speed continued to increase, the MRR could not keep up with the feeding speed of the tube electrode, resulting in short circuits. This eventually caused damage to both the workpiece and the tubular electrode, highlighting the importance of avoiding such occurrences during Laser-STEM.

### 3.5. Machining of Deep Holes and Cross-Inner Flow Channels in Titanium Alloys

**Figure 10:**
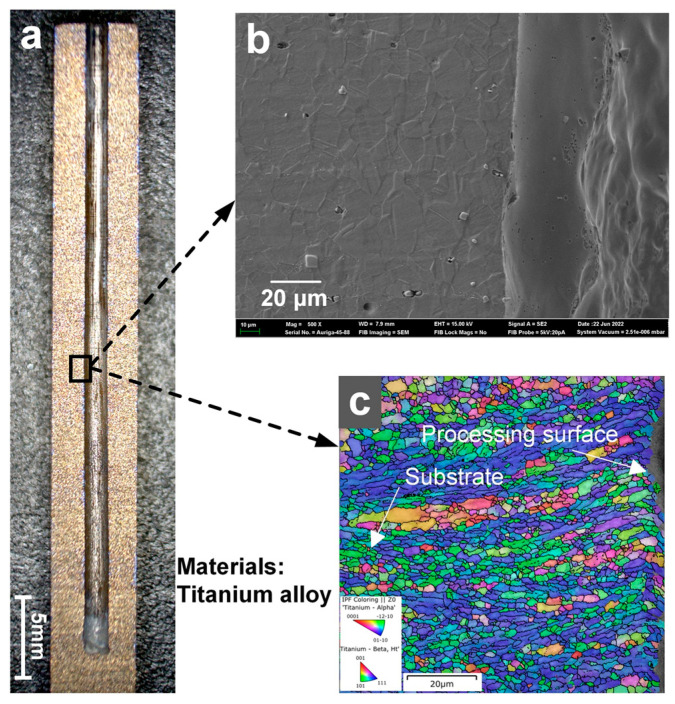
Small hole processed using Laser-STEM: (**a**) the cross-sectional profile of the small hole obtained using optical microscopy, (**b**) SEM image of the processed side wall of the small hole, (**c**) EBSD results of the processed area.

**Figure 11:**
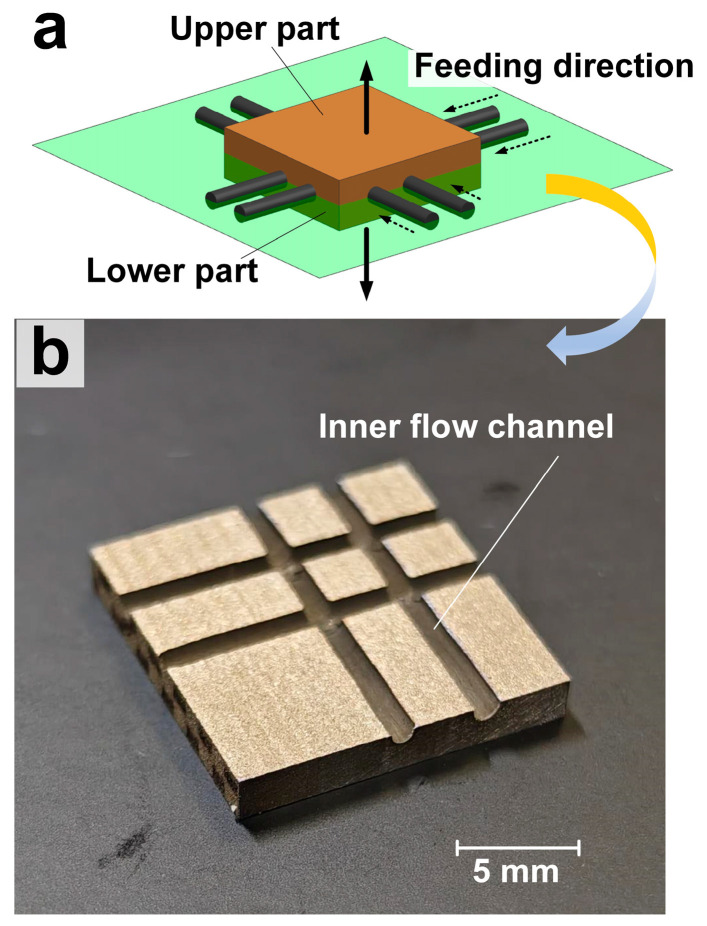
Processing of the cross-inner flow channels in titanium alloys using Laser-STEM: (**a**) schematic illustration of the process, (**b**) the cross-sectional profile of the channels.

Using Laser-STEM with a voltage of 20 V, a laser power of 5 W, and a feeding rate of 1.2 mm/min, small holes with a large depth were successfully processed in a titanium alloy workpiece. As shown in Figure 10, small holes with a diameter of 1.5 mm, a depth of 38 mm, and a surface roughness smaller than Ra 2 µm were obtained. Importantly, no short circuit or spark occurred during the machining process. The metal grains on the hole walls were analyzed using electron backscatter diffraction (EBSD), revealing a uniform distribution of grains within a field of view of about 80 µm × 80 µm with no significant difference in grain size. The grains near the hole wall were found to be oriented in the same direction of growth as the matrix grains. Notably, the grains in the entire field of view of the titanium alloy did not show any significant grain growth trend influenced by heat, such as grain enlargement. Additionally, the SEM image further confirmed that there was no recast layer and microcracks on the surface processed using Laser-STEM. Therefore, small holes can be effectively processed using Laser-STEM without the presence of a recast layer, microcracks, and heat-affected zones. As demonstrated in Figure 11, the cross-inner flow channels in titanium alloys were also processed. These channels were drilled from different directions, resulting in inner channels with a depth of 20 mm and a diameter of 1.5 mm.

## 4. Discussions

**Figure 12:**
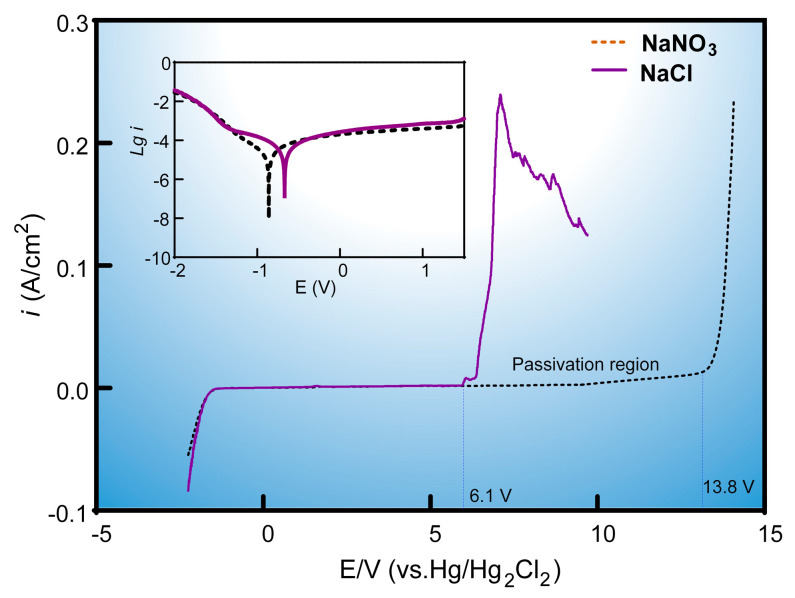
The polarization curves of titanium alloys in NaNO_3_ and NaCl electrolytes.

The results showed that the laser played distinct roles in affecting the machining precision and efficiency of Laser-STEM while processing titanium alloys with different electrolytes. To gain insights into the material removal mechanisms, the polarization curves of titanium alloys in NaCl and NaNO_3_ electrolytes were tested and subsequently analyzed. Figure 12 displays the polarization curves of titanium alloys in different electrolytes at a temperature of 18 °C and a mass fraction of 12.5 wt.%. Noticeably, the electric current density in the NaNO_3_ solution was almost zero within the voltage range of 0–13 V due to the passivation effects on the surface of titanium alloys. The passivation layer on the surface of titanium alloys could be broken with a lower voltage in NaCl electrolyte owing to the corrosion caused by chloride ions disrupting the oxide layer [29]. Meanwhile, processing titanium alloys in the NaNO_3_ solution required a higher decomposition voltage of 13.8 V compared to 6.1 V in the NaCl solution. Thus, it was concluded that the effects of the passivation layer were more pronounced when using the NaCl electrolyte during Laser-STEM.

**Figure 13:**
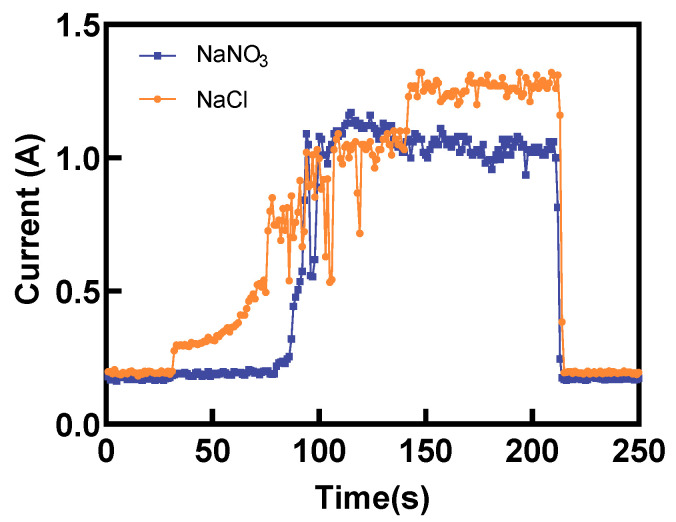
Variation of the electric current with machining time using NaCl and NaNO_3_ electrolytes during Laser-STEM.

Figure 13 presents a comparison of the electric current during Laser-STEM using NaCl and NaNO_3_ electrolytes. The electric current using NaCl increased gradually in the initial stage. Conversely, the electric current using NaNO_3_ exhibited a sudden increase when the distance between the tube electrode and the workpiece was reduced to a certain range. When utilizing NaCl as the electrolyte during Laser-STEM, the chloride ions can penetrate the passivation layer, thereby minimizing its impact on the ECM process. As the tube electrode was fed toward the workpiece, the distance between the electrode and the workpiece surface gradually decreased, leading to an increase in the electric current. Moreover, when processing titanium alloy in the NaNO_3_ solution, the resistance was mainly concentrated in the passivation layer in the initial stage. However, as the tube electrode was fed to a certain range, the voltage penetrated the passivation layer, resulting in an abrupt increase in the electric current, eventually reaching a stable processing state [30]. In addition, the electric current observed during Laser-STEM using the NaCl solution was higher than that while using the NaNO_3_ solution due to the effects of the passivation layer.

**Figure 14:**
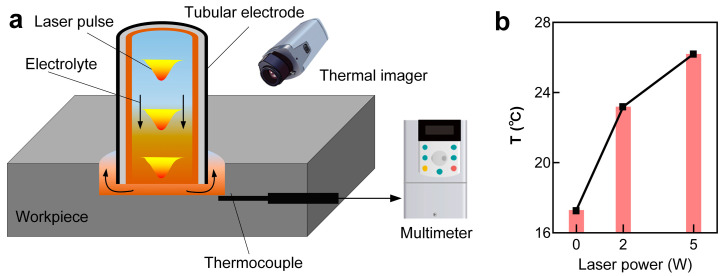
(**a**) Schematic diagram of the local electrolyte temperature measurement device during Laser-STEM, and (**b**) variation of local electrolyte temperature with laser power.

To study the impact of the laser on the temperature rise of the local electrolyte in the machining zone, the temperature of the electrolyte in proximity to the machining zone was measured using a thermocouple and thermal imager, as shown in Figure 14a. The results suggested that the local electrolyte temperature increased with the rise in laser power, as depicted in Figure 14b. Interestingly, a temperature rise of around 10 °C was observed with a laser power of 5 W. Theoretically, a higher laser power would result in a higher temperature rise in the local electrolyte.

In the process of using titanium alloys with NaNO_3_ electrolyte using ECM, the passivation layer was formed through the following reaction:

(4) $$Ti+H_{2}O\;—\to TiO_{2}+4H^{+}+4e^{-}$$

**Figure 15:**
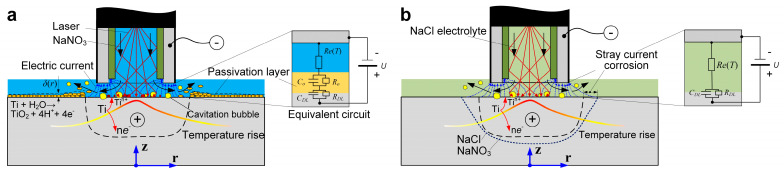
Machining models and equivalent circuits for (**a**) NaNO_3_ solution and (**b**) NaCl solution during Laser-STEM.

Further, during Laser-STEM with the NaNO_3_ electrolyte, the laser-induced temperature rise enhanced the production of the passivation layer, which was more pronounced with the increase in laser power. As shown in Figure 15b, the thickness of the oxide layer, *δ*(*r*), significantly increased with the rise in laser power. In contrast, the thickness of the passivation layer became thinner as the radius (*r*) increased, owing to the distribution of the laser intensity on the workpiece surface. Under these conditions, the resistance of the passivation layer (*R*_0_) on the surface of the titanium alloys increased with the rise in laser power. Consequently, the machining side gap was decreased due to the reduced effects of stray corrosion, resulting in an improvement in the machining precision of Laser-STEM with an increase in laser power. These findings align with the experimental results presented in Section 3.2.

**Figure 16:**
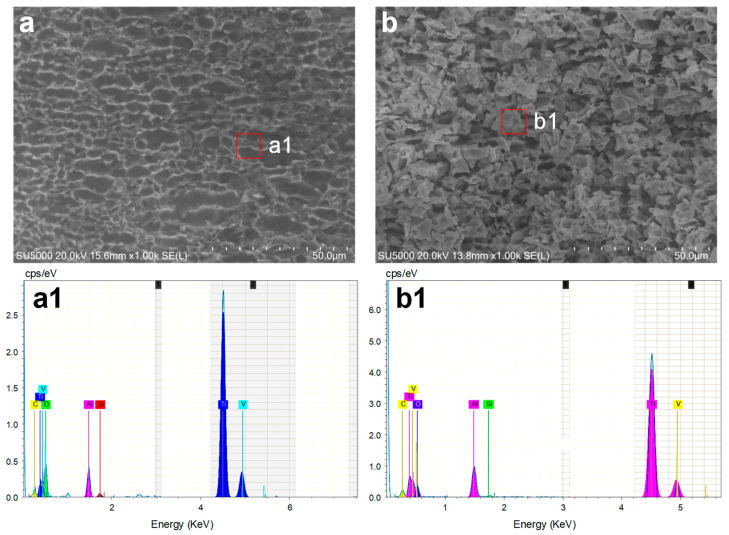
SEM and EDS results of the surface processed using Laser-STEM using (**a**,**a1**) the NaCl electrolyte and (**b**,**b1**) the NaNO_3_ electrolyte at a laser power of 5 W.

Furthermore, when using the NaCl solution as the electrolyte, the increased local electrolyte temperature facilitated the movement rate of chloride ions, enhancing the removal rate of the oxide layer [31]. Meanwhile, the electrolyte resistance (*R*e(*T*)) decreased with the increase in the local temperature of the electrolyte, as shown in Figure 15b. Moreover, the MRR and machining gap showed an increase with the rise in laser power, consequently deteriorating the machining precision of Laser-STEM using the NaCl electrolyte. At the same time, the absence of a passivation layer to stop stray current corrosion resulted in a broader range of stray corrosion in the NaCl solution. Thus, due to the resistance of the oxide layer, the electric current was higher in the NaCl solution than that using the NaNO_3_ electrolyte. Figure 16 illustrates the comparison of the machined surfaces obtained using Laser-STEM. The surface processed with NaCl electrolyte exhibited numerous micro pits caused by the corrosion of chloride ions. On the contrary, the surface processed with the NaNO_3_ electrolyte was covered with an oxide layer. In conclusion, the use of the NaNO_3_ electrolyte during Laser-STEM enhanced the machining precision with increasing laser power, whereas the use of the NaCl electrolyte improved machining efficiency.

## 5. Conclusions

In this work, the machining performance of Laser-STEM using two different types of electrolytes was compared, and the corresponding material removal mechanisms were studied. The effects of laser power and voltage on MRR, surface roughness, and the machining side were thoroughly investigated. The study also explored the maximum feeding rate of the tubular tool electrode using different electrolytes. Additionally, the polarization curve and local electrolyte temperature were measured to reveal the material removal mechanisms. Based on the findings, the following conclusions can be drawn:

(1) The laser exerted both a temperature-increasing effect and directly removed material, leading to a substantial increase in MRR with laser powers of 3–5 W. Specifically, the MRR with the NaCl electrolyte increased from 3.99 mg/min to 4.65 mg/min. Meanwhile, when using the NaNO_3_ electrolyte in Laser-STEM, the laser-induced thermal effects promoted the formation of the passivation layer on the surface of the titanium alloys, reducing the stray current corrosion and enhancing the machining precision of ECM. As a result, the side gap with the NaNO_3_ solution decreased from 90.66 µm to 73.08 µm as the laser power was increased from 0 W to 5 W.

(2) With a voltage of 20 V and a laser power of 4 W, the maximum processing speed of Laser-STEM using the NaCl solution reached 6.0 mm/min; meanwhile, the maximum processing speed was 3 mm/min with the NaNO_3_ solution. These values were increased by 200% and 333%, respectively, compared to the maximum processing speed without laser action.

(3) The study achieved small holes with a diameter of 1.5 mm, a depth of 38 mm, and a surface roughness below Ra 2 µm. Moreover, the cross-inner flow channels in the titanium alloys were also effectively processed. The SEM and EBSD results demonstrated that the processed surface showed no presence of a recast layer, microcracks, or heat-affected zones.

(4) The study further utilized polarization curves and measured local electrolyte temperature to reveal the material removal mechanisms in different electrolytes during Laser-STEM. A model was established to illustrate these mechanisms. The laser-induced thermal effect was found to enhance the movement rate of ions within the electrolyte, resulting in a reduced equivalent resistance of the electrolyte and an increased processing current. When using the NaNO_3_ solution, the laser-induced thermal effects led to an increased passivation layer thickness, thereby improving the machining accuracy of Laser-STEM. On the other hand, with the NaCl solution, the increased electric current caused by the laser-induced thermal effects enhanced the MRR of Laser-STEM.

## Data Availability

Data are contained within the article.
